# Assessing research self-efficacy among primary health care physicians: a snapshot from Qatar

**DOI:** 10.1186/s12875-022-01717-8

**Published:** 2022-05-06

**Authors:** Iheb Bougmiza, Sarah Naja, Mohamad Alchawa, Muna Abed Alah, Noora Al Kaabi, Noora Al Kubaisi, Nagah Selim

**Affiliations:** 1 Primary Health Care Corporation, Doha, Qatar; 2 Hamad Medical Corporation, Doha, Qatar

**Keywords:** Research self-efficacy, Primary health Care physicians, Qatar

## Abstract

**Background:**

Research self-efficacy is one of the crucial predictors of productively engaging in research activities emphasized by the Qatar National Vision 2030. Nevertheless, studies typically focus on research self-efficacy among students, neglecting physicians, despite the importance of research as competency in continuous professional development. Therefore, the objective of our study is to understand the level of research self-efficacy among physicians and its determinants.

**Methods:**

An analytical cross-sectional design was employed. We utilized an open survey through DACIMA Software that included questions related to Self-Efficacy in Research Measure (SERM) and possible determinants. One-hundred-twenty-two completed answers, and the response rate was 19.2%. Following descriptive analyses, a chi-square test was used to uncover the associations among variables, with significance set to *p* ≤ 0.05. Next, a logistic regression model was conducted to identify the predictors of a low research self-efficacy level. Finally, reliability and principal component analysis were applied on the SERM scale.

**Results:**

Three-quarters of the sample reported insufficient research self-efficacy. The sociodemographic and professional factors did not significantly associate with insufficient research self-efficacy. However, participation in clinical guidelines proved to be a determinant of sufficient research self-efficacy.

**Conclusions:**

Physicians must be encouraged to participate in clinical guidelines to improve their research self-efficacy level.

## Introduction

Self-efficacy is a critical element in social cognitive theory and a social foundation of thoughts and action, defined by Bandura as “The belief in one’s capabilities to accomplish a certain level of performance” [1]. This belief depends on psychosocial interactions between four constructs: individual performance accomplishments, vicarious experience, verbal persuasion, and physiological information [2]. Sufficient research self-efficacy indicates physicians’ confidence in demonstrating research skills, enabling them to be critical of the published information and implement evidence-based medicine. Conversely, insufficient research self-efficacy distorts certainty in clinical decisions and blocks continuous professional development [3–5].

In a study conducted in the United States of America (USA) among academic physicians’ residents and fellows, self-efficacy was assessed through the online 92-item Clinical Research Appraisal Inventory. Fellows had higher self-efficacy than physicians’ residents due to their higher years of research experience [6]. Consistent results were revealed in another paper, where previous experience and mentoring are influential sources of research self-efficacy [7]. Both studies were conducted among residents still under training in their medical education path [6, 7]. Notably, the whole body of literature focuses on developing research skills among medical students and academic settings [8, 9]. However, no studies have discussed research self-efficacy among physicians in practice, even though low research self-efficacy affects generating and justifying new knowledge essential in the practice of evidence-based medicine [10].

The Qatar National Strategy prioritizes research productivity, provides funds, and considers publication one of the enabling requirements for promotion. Thus, motivating physicians to work on research [11].

No previous research addressed research self-efficacy and its determinant among Qatar’s primary healthcare physicians. We seek to address this gap by assessing research self-efficacy levels among physicians in primary health care and identifying significant determinants.

### Purpose

Examining the level of research self-efficacy among physicians working at primary health care centres (PHCC) in Qatar will enable us to understand the baseline and the determinants of low research self-efficacy to implement specific interventions in continuous professional development (CPD), which will reflect on the quality of care at primary health care level than at the health care system.

## Methods

### Ethical approval, relevant guidelines, and informed consent to participate

All methods were performed according to the relevant guidelines and regulations of Primary Health Care Corporation Research Committee approval (PHCC/RC/18/09/001).

### Study design and setting

An analytical cross-sectional design was employed among primary health care physicians. We sent a web-based survey to all physicians registered at PHCC via email with an information letter and a link to the electronic version of the questionnaire. The data collection took place from the 21st of October to the 23rd of November 2018.

PHCC provides preventive and free-of-charge services to the whole community in Qatar through 23 primary health care centres distributed across the country (total number of physicians registered at PHCC at time of data collection = 634).

### Sampling technique

We extracted the list of all the physician’s emails registered in primary health centres from primary health care operations. Again, a convenience sample was utilized, as we aimed to include all the physicians registered.

### Sample size and participants’ enrolment

Sample size calculation was not used in the study as we are using a convenience sample.

Eligible participants involved all physicians registered at primary health care who were willing to participate and communicate in English during the study period. Therefore, we did not have specific exclusion criteria.

### Data collection

The open survey was developed through DACIMA Software, an advanced web-based electronic data capture (EDC) software for collecting, managing, and reporting research data.

The survey was conducted only in English as all the physicians are knowledgeable and comfortable answering surveys and communicating in English. The PHCC research delegates emailed the physicians with an information letter and a link to the electronic version of the survey. There was no randomization of items or surveys. The average time of each participant was 15 min. We ensured data collection completion through two reminders sent each week.

The survey was advertised through the PHCC website and official emails. JavaScript checked the consistency and completeness of the submitted surveys, and it enforced one response option. The open poll did not utilize adaptive questioning. The number of items ranged between 18 to 33 per page, and the number of screen pages was 15 pages. We enabled a review check of the respondent to their answers and prevented multiple entries from the same individual through the record IP address of the individual.

### Materials

Only complete questionnaires were considered in the data analysis phase. We utilized the CHERRIES (Checklist for Reporting Results of Internet Electronic Surveys) criteria during writing the protocol [12].

We conducted piloting on 10% of the total sample that allowed testing of the usability and technical functionality of the electronic survey. Piloting did not result in any changes to the tool, so we included it in our total sample. In addition, we measured the time needed to complete each questionnaire.

The dependent variable is our primary outcome that relates to research self-efficacy among primary health care physicians [13]. This was defined as one’s ability, capabilities, and confidence to perform research [1].

Independent variables are variables that self-regulate and may have an impact or stimuli on the dependent variable [13]. The independent variables included sociodemographic characteristics and professional development factors.

### Measurements tool related to dependent variable-research self-efficacy

We utilized Self-Efficacy in Research Measure (SERM) developed by Phillips and Russell. It is a 33-item English questionnaire that measures research efficacy among professionals in various career fields. It focused on four dimensions of research self-efficacy, including (a) research design skills (8 items), (b) practical research skills (8 items), (c) quantitative and computer skills (8 items), and (d) writing skills (9 items). In addition, the respondent indicates how confident they are to perform each research: 0 (belief of inability) to 9 (belief of performing in the full item ability). The total score for each participant varies from 0 to 297. The higher the scores, the more confident the participant in the research. The Coefficient alpha of total SERM scores was reliable at 0.96 [4, 13, 14].

### Measurement tools related to independent variables

The structured questionnaire was developed through extensive literature. It included questions regarding sociodemographic (age, gender, year of graduation, and nationality) and professional development factors (clinical experience in PHCC in years, the average number of patients seen per day, speciality, academic position, computer literacy, experience developing the clinical guideline, previous training in research methodology, and previous publications).

Computer literacy was assessed through one question (Not at all, basic, good, excellent), and self-efficacy in using guidelines was assessed through five items, and it was questioning confidence in utilizing guidelines (I strongly agree, I disagree, I strongly disagree).

An expert panel established the questionnaire’s face validity (English) and relevance to the study objectives. Additionally, we verified the content validity through an extensive literature review to ensure the consistency of the contents and scale level. Finally, using Lawshe’s method, each item was rated for its importance and relevance by applying a three-point scale: (1) not necessary, (2) useful but not essential, and (3) essential. The universal agreement between the three evaluators was 80%.

### Analysis

We analyzed data through Statistical Package for the Social Sciences (SPSS), Version 20.0 (IBM Corp, Chicago, Illinois, USA). The statistical analysis involved descriptive summarization of the variables: categorical variables in frequency and percentages and continuous variables in mean ± standard deviation (SD).

We conducted a bivariate analysis to test associations between dependent and independent variables through Pearson’s chi-squared test (odds ratio (OR) and 95% CI). In addition, we included all determinants in multivariable logistic regression analysis. We computed the adjusted odds ratio (entry method Logistic Regression). We also performed reliability testing and principal component analysis of the ‘Self-Efficacy in Research Measure’ (SERM) tool.

The Domain score was calculated by summing all feasible items in the domain named Index. The Index is the summation of all the scores of each item and multiplying it by a total number of items, 33, then dividing them by 297 (which is the highest code, 9, multiplied by the number of items, 33). We then multiplied the Index by 100. A standardized score was obtained to the total score, then cut-off points of 75 percentile discriminated high self-efficacy from low-self efficacy; several papers inspire domain calculation [15–17].

*P*-values ≤ 0.05 were considered statistically significant. As data were collected first in Microsoft Excel and then extracted to SPSS, to ensure the quality of data entered, we performed an audit on 10% of the data entered by another researcher.

## Results

### Sample realization

**Fig. 1:**
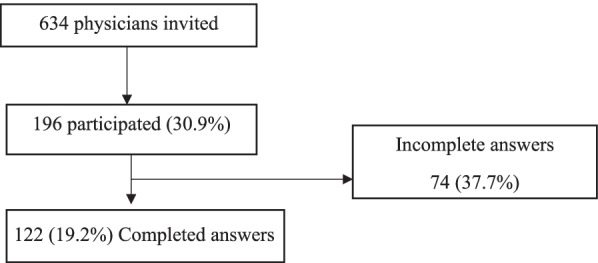
Flow chart of participants (*n* = 196)

We invited 634 physicians during this period to participate in the study. Less than half of the physicians agreed to complete the survey (*n* = 196, 30.9%). Only 122 (19.2%) provided a completed survey, as seen in the flowchart in Fig. 1.

### Participant characteristics

The age of the enrolled participants averaged 41 years ± 7.9 and ranged between 25 and 62 years. Most of the participants were male (*n* = 77, 63.1%) and non-Qatari (*n* = 107, 89.2%). The majority reported good (*n* = 71, 60.7%) to excellent (*n* = 41, 35%) computer literacy level. Family medicine practitioners (*n* = 106, 86.9%) had the highest distribution; the average yearly graduation was in 2001. The mean of employment at PHCC was 6 years ± 6.8. It is worth mentioning that most physicians did not publish articles (*n* = 90, 73.8%).

### Prevalence of insufficient research self-efficacy

Of the 122 participants, almost three-quarters (*n* = 92, 75.4%) showed insufficient research self-efficacy, and one-quarter (*n* = 30, 24.6%) demonstrated sufficient research self-efficacy. The mean scores of the subscales and items are reported in Table 1.

**Table 1:** Research self-efficacy score among physicians in primary care in Qatar, 2018 (*n* = 122)

Subscale and items	Mean (SD)	Range
**Research design skills**	**25.4 (17.7)**	**65**
1. Selecting a suitable topic for study	**5.7 (2.5)**	**9**
2. Designing an experiment using non-traditional methods (e.g., ethnographic, cybernetic, phenomenological approaches)	**3.9 (2.5)**	**9**
3. Designing an experiment using traditional methods (e.g., experimental, quasi-experimental designs)	**4.6 (2.7)**	**9**
4. Controlling for threats to validity	**3.7 (2.7)**	**9**
5. Formulating hypothèses	**3.5 (2.7)**	**9**
6. Selecting a sample of subjects from a given population	**3.4 (2.8)**	**9**
7. Selecting reliable and valid instruments	**3.5 (2.7)**	**9**
8. Operationalizing variables of interest	**3.8 (2.8)**	**9**
**Practical research skills**	**27 (18.8)**	**67**
9. Getting an adequate number of subjects	**4.5 (2.9)**	**9**
10. Keeping records during a research project	**2.5 (2.4)**	**9**
11. Collecting data	**2.9 (2.6)**	**9**
12. Making time for research	**3.1 (2.7)**	**9**
13. Contacting researchers currently working in an area of research interest	**3.6 (2.8)**	**9**
14. Utilizing resources for needed help	**3.9 (2.9)**	**9**
15. Defending a thesis or dissertation	**3.5 (2.8)**	**9**
16. Getting money to help pay for research	**3.1 (2.7)**	**9**
**Quantitative and computer skills**	**24 (18.0)**	**65**
17. Knowing which statistics to use	**3.0 (2.7)**	**9**
18. Manipulating data to get it onto a computer system	**3.2 (2.7)**	**9**
19. Avoiding the violation of statistical assumptions	**2.9 (2.7)**	**9**
20. Using simple statistics (e.g., t-test, ANOVA, correlation, etc.)	**3.2 (2.9)**	**9**
21. Understanding computer printouts	**2.7 (2.6)**	**9**
22. Using multivariate statistics (e.g., multiple regression, factor analysis, etc.)	**3.1 (2.8)**	**9**
23. Using statistical packages (e.g., SPSS-X, SAS, etc.)	**2.9 (2.8)**	**9**
24. Writing statistical computer programs	**3.4 (2.9)**	**9**
**Writing skills**	**31.4 (23.1)**	**79**
25. Writing a research presentation for a conference	**3.7 (2.9)**	**9**
26. Writing the method and results section for a research paper for publication	**3.0 (2.8)**	**9**
27. Writing a discussion section for a thesis or dissertation	**2.4 (2.5)**	**9**
28. Writing the introduction and literature review for a dissertation	**2.3 (2.7)**	**9**
29. Reviewing the literature in an area of research interest	**3.1 (2.7)**	**9**
30. Writing the introduction and discussion sections for a research paper for publication	**2.8 (2.6)**	**9**
31. Writing the method and results sections of a dissertation	**2.1 (2.4)**	**9**
32. Writing the introduction and literature review for a thesis	**2.1 (2.4)**	**9**
33. Writing the method and results sections of a thesis	**2.3 (2.5)**	**9**

*SD* Standard deviation

### Determinants of insufficient research self-efficacy

The result showed that previous participation in developing clinical practice guidelines increases the likelihood of sufficient research self-efficacy by threefold. Nationality, age, and previous publications did not show any significant association with research self-efficacy (Table 2).

**Table 2:** Sociodemographic and professional factors associated with sufficient and insufficient self-efficacy among primary health care physicians in Qatar, 2018 (*n* = 122)

Sociodemographic Characteristics	Research Self-efficacy Levels						
	**Insufficient (< score 55.5)**		**Sufficient (≥ score 55.5)**				
	***N***	**(%)**	***N***	**(%)**	**χ**^**2**^	**OR,95%CI**	***P***
**Nationality**							
Qatari	10	(10.9)	3	(10.3)	0.006^a^	1.1 [0.2–4.1]	0.9
Non-Qatari	82	(89.1)	26	(89.7)			
**Gender**							
Male	57	(62)	20	(66.7)	0.21^a^	1.2 [0.5–2.9]	0.61
Female	35	(38)	10	(33.3)			
Participation in the development of CPGs *							
Yes	14	(15.2)	12	(40)	8.2	3.7 [1.4–9.3]	0.004*
No	78	(84.8)	18	(60)			
**Review of CPGs**							
Yes	19	(21.6)	10	(33.3)	1.6	1.8 [0.7–4.5]	**0.19**
No	69	(78.4)	20	(66.7)			
**Training on research methodology**							
Yes	40	(45.5)	19	(63.3)	2.8	2.1 [0.8–4.8]	0.09
No	48	(54.5)	11	(36.7)			
**Previous publication in indexed journals**							
Yes	22	(25)	12	(40)	2.4	2 [0.8–4.8]	**0.11**
No	66	(75)	18	(60)			
**Self-efficacy in using EBM**							
Yes ^(> 75 percentile)^	23	(25)	12	(40)	2.4	2[0.8–4.7]	**0.11**
No ^(< 75 percentile)^	69	(75)	18	(60)			

CPGs Clinical Practice Guideline, EBM Evidence Based Medicine, * p ≤ *0.05 /* χ^2**=**^*Chi-square/ a* = *Fisher Test/OR* = *Odd Ratio*

The multivariate model showed that participation in developing guidelines is the only predictor for research self-efficacy. All other factors, such as age, gender, clinical experience, nationality, speciality, and training in methodology, failed to predict the outcome (Table 3).

**Table 3:** Multivariable analysis of the predictors of sufficient vs insufficient self-efficacy among primary health care physicians in Qatar, 2018 (*n* = 122)

Variables	Variables in the equation Research self-efficacy						
						95% CI for aOR	
	B	S. E	Wald	Sig	aOR	Lower	Upper
**Gender**							
Male	-0.09	0.52	0.032	0.85	0.91	0.32	2.55
Female					1		
**Nationality**							
Qatari	0.66	0.81	0.67	0.41	1.9	0.39	9.5
Non-Qatari					1		
Participation in the development of CPGs *							
Yes	1.56	0.80	3.7	0.04*	4.7	1.99	22.0
No	1.56	0.80	3.7	0.04*	1		
**Review of CPGs**							
Yes	-0.78	0.79	0.96	0.32	0.45	0.09	2.11
No					1		
**Training on research methodology**							
Yes	0.48	0.51	0.89	0.34	1.62	0.59	4.42
No					1		
**Previous publication in indexed journals**							
Yes	0.47	0.51	0.85	0.35	1.61	0.58	4.44
No					1		
**Self-efficacy in using EBM**							
Yes ^(> 75 percentile)^	0.31	0.50	0.39	0.53	1.37	0.50	3.70
No ^(< 75 percentile)^					1		

*B: B coefficient, aOR*  Adjusted Odd Ratio, *P value ≤ *0.05; CI* = *Confidence Interval*

The model was obtained using entry selection

## Discussion

This study investigated research self-efficacy among physicians working in primary health care clinics in Qatar. Almost three-quarters (*n* = 92, 75.4%) showed insufficient research self-efficacy. In addition, a significant association was found between insufficient research self-efficacy level and lack of participation in clinical guideline development.

Our results reveal that the factor’ participation in clinical guidelines’ increases the likelihood of sufficient research self-efficacy by threefold. Furthermore, the logistic regression model proved that this factor significantly predicts insufficient research self-efficacy. Thus, recommending participation in clinical guidelines will positively reflect their research self-efficacy level and eventually lead to better practice.

Gender was a non-significant factor. This finding is consistent with another study conducted among physicians [6].

We reported total scales rather than the subscales as, in a previous publication, the confirmatory factor analyses did not support the sub-analysis [13].

This research is the first to examine research self-efficacy status among physicians in the Arab Gulf region. The regional body of research has focused on medical students only. The strength of this study is that it aims to explore the research skills of primary care physicians. Research competency is an essential skill for physicians as it contributes to evidence-based medicine and improves patient care. It is an original topic that evaluates physicians’ research skills and allows them to develop research capacity-building activities.

The ‘Self-Efficacy in Research Measure (SERM)’ is a valid instrument. In addition, we delivered evidence for a three-factor structure tool (short version) that could be used in time-critical situations for physicians to ensure high response rates.

Several strategies were employed to decrease the measurement bias, including selecting a reliable tool (SERM) with good internal consistency (Cronbach’s alpha = 0.98). Similarly, the content and face validity of the questionnaire were assured through appropriate techniques. The CHERRIES (Checklist for Reporting Results of Internet Electronic Surveys) criteria were utilized in reporting.

The study’s cross-sectional design compromises causation as it specifically lacks temporality. Furthermore, external validity is affected by utilizing a non-probability sampling technique. Fortunately, the selected sample revealed heterogeneity and included variable sociodemographic subgroups, reflecting positively on the population’s accurate account [18]. However, another limitation was that the survey was long, leading to a low sample size that affects external validity and generalizability with a response rate of 19.2%. 

## Conclusion

This study indicates that insufficient research self-efficacy is common among physicians in Qatar. Therefore, health officials should design and implement targeted interventions to promote research self-efficacy by mandating physicians to be trained in developing clinical guidelines. The next step would be a confirmatory, controlled study. Once that is done, firm recommendations might be available.

## Data Availability

The datasets used and/or analysed during the current study are available from the corresponding author on reasonable request.

## Abbreviations

SERM: Self-Efficacy in Research Measure
CHERRIES: Checklist for Reporting Results of Internet Electronic Surveys
EBM: Evidence-Based Medicine
CPGs: Clinical Practice Guideline
χ^2^: Chi-square
a: Fisher Test
CI: Confidence Interval
OR: Odd Ratio
